# Supplementary material for: Comparison of Quality, Antioxidant Capacity, and Anti-Inflammatory Activity of Adlay [Coix lacryma-jobi L. var. ma-yuen (Rom. Caill.) Stapf.] Sprout at Several Harvest Time
Source: Plants (Basel). 2023 Aug 17;12(16):2975. doi: 10.3390/plants12162975 (PMC10458144; doi:10.3390/plants12162975)
Supplement: Supplementary file 1 [file plants-12-02975-s001.zip › plants-2351727-supplementary.pdf]

Supplementary Material

Table S1. Production mass increase index according to the harvest time.

Days after sowing	Production mass (g/cell)	Increase index
3 days	161.4 e	-
5 days	447.8 d	2.8
7 days	814.0 c	1.8
9 days	1100.9 b	1.4
11 days	1319.1 a	1.2

Table S2. Comparison of production mass per cell harvest at 7th day after sowing according to the light and dark condition.

Light condition	Production mass (g/cell)
Light (24h MH lamp)	814.0 a
16 light / 8 dark	769.2 ab
Dark	745.7 b

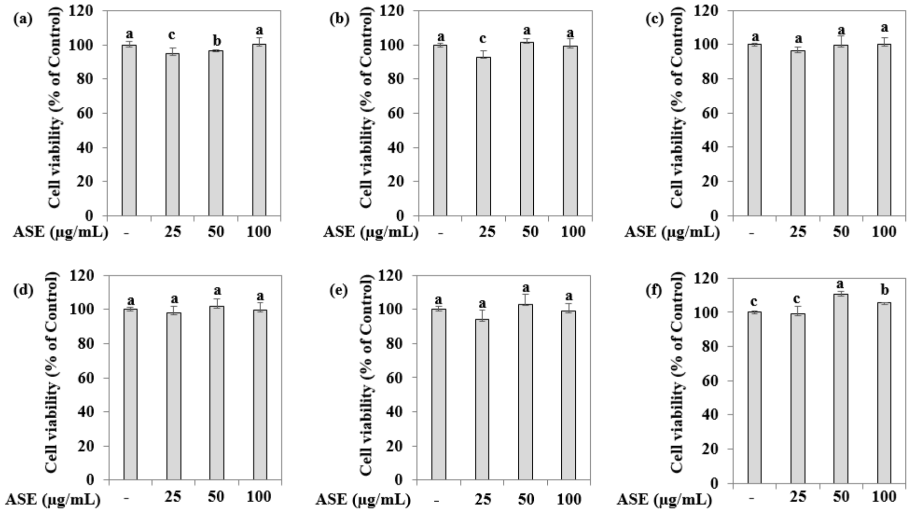

Figure S1. Cytotoxic effects of adlay sprout extract (ASE) against H_2O_2-induced oxidative stress in HepG2 cells. (a) Adlay sprouts extract of 0 day after sowing; (b) Adlay sprouts extract of 3 days after sowing; (c) Adlay sprouts extract of 5 days after sowing; (d) Adlay sprouts extract of 7 days after sowing; (e) Adlay sprouts extract of 9 days after sowing; (f) Adlay sprouts extract of 11 days after sowing. HepG2 cells were exposed to various concentrations of adlay sprouts extract and hydrogen peroxide (1 mM H_2O_2) for 24 h. The cell viability was determined by WST-1 assay. Control, group of non-treated samples; H_2O_2, group of treatment with 1 mM hydrogen peroxide. Statistically significantly different letters when compared with each extract included control and H_2O_2 ($P<0.05$, one-way ANOVA).

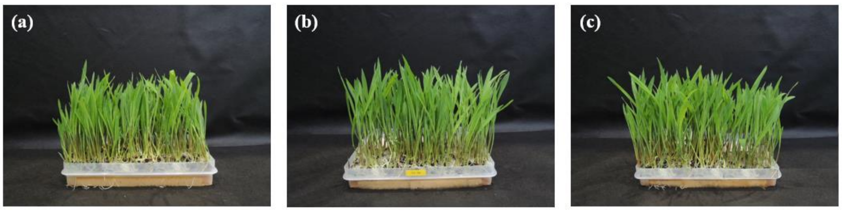

Figure S2. Growth of adlay sprouts according to the irrigation frequency per day. (a) Irrigation once a day; (b) Irrigation 3 times per day; (c) Irrigation 5 times per day.

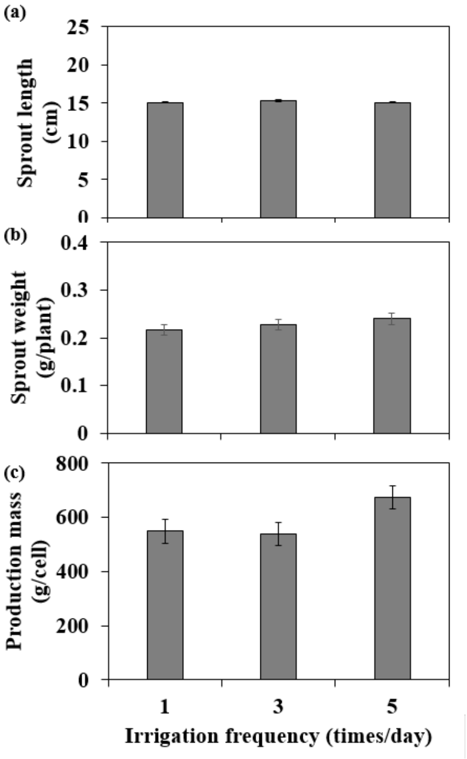

Figure S3. Sprout length, weight and production mass of adlay sprouts according to the irrigation frequency per day. (a) Sprout length; (b) Sprout weight per plant; (c) Production mass per cell(60x30cm).

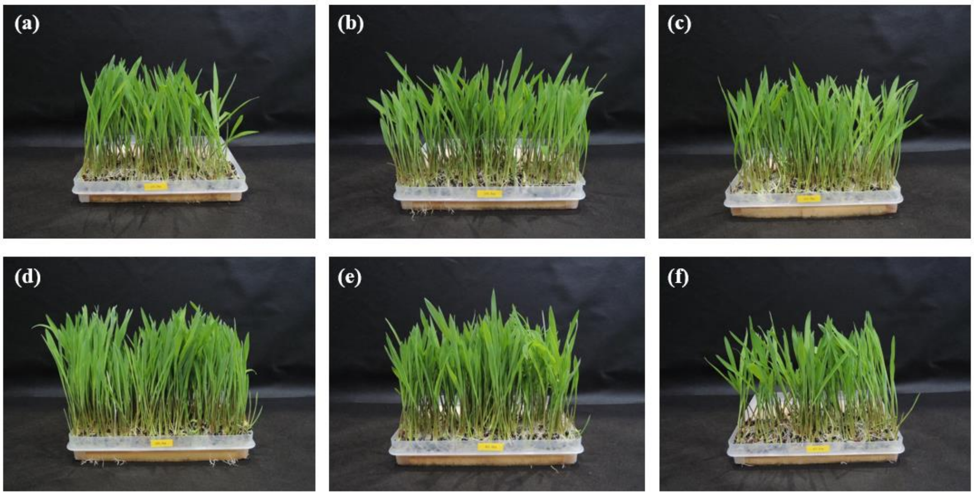

Figure S4. Growth of adlay sprouts according to the seeding density. (a) 500g; (b) 600g; (c) 700g; (d) 800g; (e) 900g and (f) 1,000g per cell(60x30cm).

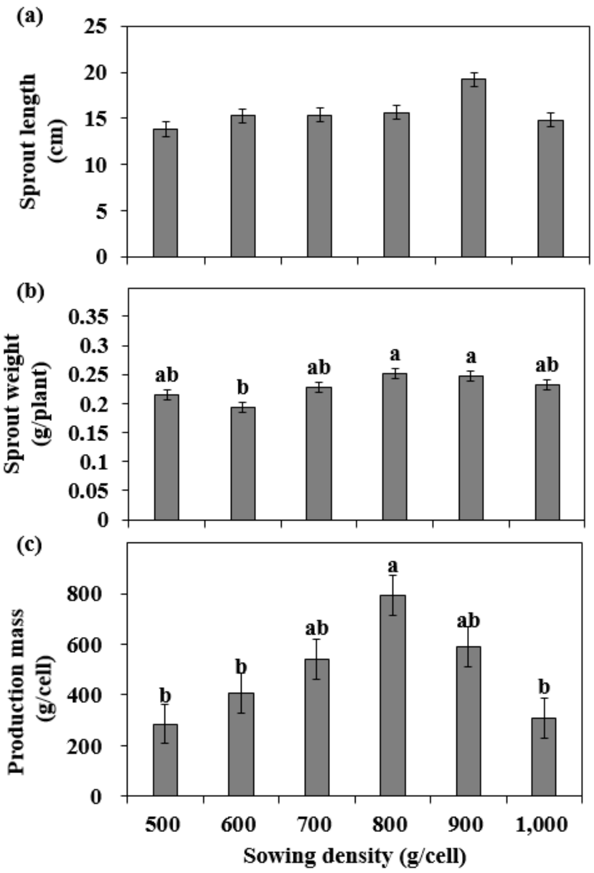

Figure S5. Sprout length, weight and production mass of adlay sprouts according to the seeding density. (a) Sprout length; (b) Sprout weight per plant; (c) Sprout yield per cell(60x30cm).
